# Rapid Automatized Naming (RAN) and Word Reading Fluency in Early School-Aged Children: A Pilot Eye-Tracking Study

**DOI:** 10.3390/jemr19010016

**Published:** 2026-02-04

**Authors:** Alisa Baron, Alexia Martins, Gavino Puggioni, Vanessa Harwood

**Affiliations:** 1 Department of Communicative Disorders, The University of Rhode Island, Kingston, RI 02881, USA; alexia.pariseau@gmail.com (A.M.); vharwood@uri.edu (V.H.); 2 Department of Computer Science and Statistics, The University of Rhode Island, Kingston, RI 02881, USA; gpuggioni@uri.edu

**Keywords:** rapid automatized naming, eye tracking, children, reading

## Abstract

Fluent word reading is a key literacy skill, yet the full extent of the oculomotor underpinnings in developing readers remains unknown. Rapid automatized naming (RAN) is a useful clinical measure that has been shown to predict word reading fluency. Here we use RAN scores to predict early, mid, and late local stages of word reading as measured by eye tracking in children who are at a critical time in their literacy development. Thirty-three children participated in two RAN tasks (rapid letter naming (RLN) and rapid digit naming (RDN)) and an eye-tracking task, which included sentence-level reading with an embedded target word. The eye-tracking measures of first fixation duration, regression path duration, and total word reading time were used as early, mid, and late local measures, respectively. RLN and RDN significantly predicted only the mid-stage of the reading process (regression path duration). Faster RLN and RDN times were associated with briefer regressions from target words. Preliminary results link behavioral RAN performance to a mid-stage oculomotor variable, indicating that children with slower RAN times may exhibit longer regressions during reading, suggesting possible difficulties with the integration of phonological processing skills.

## 1. Introduction

Phonological processing skills play a critical role in the development of reading and writing. They are widely evaluated in young children as they are predictive of word reading abilities [1]. Phonological processing is an umbrella term which includes the ability to analyze, recall, and manipulate phonological units [2]. Based on Wagner’s model of phonological processing, there are three main components of phonological processing closely related to decoding abilities that are often assessed in children: phonological awareness, phonological working memory, and rapid automatized naming (RAN), i.e., [1,3,4]. Phonological awareness and phonological working memory are primarily auditory-based tasks. However, RAN requires the integration of both auditory and visual information, making it highly similar to the reading process [5].

RAN is often cited as a measure of *lexical retrieval* [1,6,7,8,9]. Lexical retrieval involves the process of retrieving a specific word from the lexicon, including its phonological and articulatory information, and using it in speech or writing. One important component of lexical retrieval is lexical access, which involves the processes associated with retrieving the word and phonological information associated with the word; however, lexical access may not involve the articulatory production of the word. Indeed, the requirements of RAN tap several cognitive processes that parallel what is necessary when children read text, including attention to the stimulus, initial feature detection, visual discrimination, rapid integration of visual symbols, associating symbols with their phonological components, integration of patterned information, access and retrieval of phonological labels, and lexical recall [5,10]. Wolf, Bowers, and colleagues (1999) have therefore emphasized the “orthographic account” for RAN, as it integrates both visual and linguistic properties that are particularly important for the development of orthographic representations [11]. RAN has been described as a “microcosm” of reading given that the neural networks responsible for RAN performance are shared more broadly with general reading performance, e.g., [12,13,14]. Consequently, RAN acts as a strong predictor of current and future reading abilities [11,15]. Therefore, RAN becomes an important assessment tool as children move from early decoders to more fluent readers.

### 1.1. Associations Between RAN and Reading

Successful RAN performance depends on several factors. Gordon and Hoedemaker (2016), using eye-tracking technology, concluded that response times to serial alphanumeric stimuli were more strongly associated with automaticity and the encoding-to-articulation process during reading [16]. This effect may be attributed to the limited and well-defined sets from which letters and digits are selected. Further, several studies have suggested that RAN performance is influenced by age and instruction. Kirby et al. (2003) suggested that naming speed best predicts reading skills in later years, possibly due to the change in reading from phonetic to orthographic processes [15]. Similarly, both Hogan (2005) and Wolff (2014) reported that explicit phonics training (i.e., training in the alphabetic principle, the relationship between letters and spoken sounds) played a role in increasing RAN’s reliability in predicting reading fluency in later years [1,17]. However, a more recent meta-analysis of RAN in preschoolers and kindergarteners suggested that RAN performance is related to future word reading in this age group, similar to findings for readers of various ages and languages [18]. Although many studies have focused on auditory measures of phonological processing, RAN has been reported to account for variance in word reading ability over and above phonological awareness and phonological working memory [16,19,20,21]. Therefore, RAN performance can be seen as a highly sensitive, ecologically sound predictor of reading performance worthy of investigation.

RAN has been associated with skills such as reading accuracy and reading fluency, which are often the focus of literacy assessments for school-aged children as they are necessary components of skilled reading [10]. Haager et al. (2014) define reading accuracy as the ability to recognize words automatically or with little effort in decoding, as children encounter more difficult words, decoding can and does become more effortful [22]. Reading fluency is the ability to decode with “speed and quality, involving minimal conscious effort from cognitive and linguistic processes” [5] (p. 429). Reading fluency involves reading with appropriate pace, expression (prosody), and phrasing to convey meaning in text. The combination of both reading accuracy and fluency is critical in supporting adequate reading of text and text comprehension [23].

RAN tasks can vary in terms of their design and items. Stimuli within a RAN task can be presented in a left-to-right array, or in a right-to-left array, depending on the orthography (i.e., serial RAN), or one at a time (i.e., discrete naming; [24]). Although naming speed has been assessed in both formats, discrete naming tasks are inconsistent predictors of reading skills, whereas serial RAN tasks are strongly related to reading fluency [25,26,27]. Serial RAN allows for greater association between the typical left-to-right manner of reading in English, which requires sustained attention and consistent visual tracking of text. RAN tasks also vary in terms of their items. The rapid naming of letters, digits, colors, and objects has been associated with literacy performance [28]. However, Araújo et al. (2015) concluded that alphanumeric stimuli (letters and digits) correlate best with reading fluency rather than non-alphanumeric stimuli (colors and objects), e.g., [29,30,31]. Tasks with alphanumeric stimuli are performed faster and become automatized earlier in development, e.g., [32,33,34,35,36], possibly due to the explicit serial nature in which letters and numbers are taught. Although rapid letter naming and rapid digit naming are highly correlated and tend to group together in larger factor analyses [37,38], there are some discrepancies in performance between the two tasks as they may capture different processing skills. Rapid letter naming has a stronger correlation with reading abilities compared to digit RAN tasks. This is because letter naming directly engages the same cognitive processes involved in reading, such as letter recognition and phonological processing [39,40]. While still related to reading, digit naming may not engage phonological processing in the same way as letter naming, resulting in a weaker correlation with reading skills [30]. Letter names may be more easily confused with their letter sounds. Digits may be learned earlier, and there are significantly more letters than digits, making letter knowledge more discriminating. Although the various naming tasks are interrelated, the factors are not as clearly identified at younger ages [38]. Thus, further investigation of each naming task separately in younger children is still warranted.

Alphanumeric stimuli are strongly related to word reading performance in both typically developing children as well as clinical populations. Poor RAN performance has been noted in children with reading disability (RD), children with Developmental Language Disorder (DLD), and children with co-morbid RD and DLD [19]. Interestingly, children who do not present with an RD but do qualify as having DLD may perform poorly on RAN tasks yet demonstrate intact word reading abilities. It is possible that some children with DLD develop compensatory orthographic strategies at the text level, leading to intact word reading abilities [41]. The fact that RAN deficits are seen in both RD and DLD populations highlights RAN’s clinical utility as a sensitive indicator of impairment. RAN has also been highly investigated in multilingual learners. Recent studies indicate that RAN remains a reliable predictor of reading fluency in multilingual children, reflecting the efficiency of lexical retrieval and automaticity processes that support word recognition across languages [42]. Although the strength of the RAN–reading relationship may vary with language proficiency and orthographic depth of the language, RAN contributes uniquely to decoding and reading speed beyond phonological awareness in multilingual learners [43,44]. Collectively, these studies suggest that RAN’s high clinical utility makes it a sensitive measure of skill across several populations.

### 1.2. Eye Tracking as a Method to Measure Reading Fluency

The literature regarding the RAN–reading relationship has focused on the conceptualization [45] and assessment [26] of RAN as there is a strong relationship between the two across the lifespan [15,25,46,47,48]. Reading accuracy and fluency are critical skills, yet their oculomotor underpinnings remain poorly understood due to the complexity of processes that underlie how children learn to read. Eye-tracking technology is a powerful tool that can allow for a more fine-grained, natural, and quantifiable record of word reading behavior in comparison to traditional behavioral measures of reading [49]. Eye tracking provides detailed parameters of visual word processing and a potential means to identify the underlying cognitive processes associated with word reading.

Recently, due to the use of eye-tracking methods, the focus has switched from explaining the role of RAN in predicting reading [50] to uncovering RANs underlying factors [14,51]. Studies utilizing eye tracking to measure word reading behaviors have been used extensively with adults; however, far fewer have used eye tracking to investigate aspects of reading in the school-aged population, e.g., [14,50,52,53,54]. The standard behavioral approach for RAN only provides a single time point to obtain accuracy in a dichotomous way (correct or incorrect), meaning that the internal cognitive stages during which informational processing occurs during reading are disregarded or understudied [55]. Therefore, eye tracking may provide significant insight into the development of word-reading skills at a critical time of the learning process.

Despite eye tracking being a widely used tool in reading research, there remains a great amount of variability in methodological procedures, particularly in the measures used to capture different aspects of eye movements related to text reading. Given the vast measures available, it is imperative that reading researchers clearly define their variables and justify which measures most closely align with the theoretical basis of their research questions [56]. Godfroid (2020) conducted a synthetic review of eye-tracking studies and found that there are four reading duration measures (“big four”) that have become standard in the adult text-based eye-tracking literature [57]. These big four measures include first fixation duration, gaze duration, regression path duration, and total reading time. In-depth definitions of each measure are included within the Current Study Section.

Eye-tracking measures can also be divided into early, mid-, and late-stage measures of the reading process. Early measures appear to index processes that occur in the initial stages of word or sentence processing, such as word recognition and lexical access [57,58,59]. Longer fixation durations of less skilled readers may indicate that these readers extract less orthographic information per fixation from very simple stimuli [52]. Late measures, however, may signal an interruption [57] to the normal reading process and therefore may index more labored reading. A regression is considered a late-stage measure as it may indicate that a child has to re-read sentence parts that were not fully processed during the initial reading of text. Regressions can be present when there may be difficulties in the simultaneous processing of both orthography and syntax, and therefore slow down the reading process. However, it is important to note that regressions can represent labored reading but also may indicate skillful checking for more difficult, ambiguous text for older students as they become more skilled at monitoring comprehension. Therefore, careful classification of reading behaviors through the use of eye tracking and their relationships with different aspects of reading performance is essential in deepening our understanding of successful reading performance in early school-aged children.

### 1.3. Current Study

RAN is a highly used ecologically valid predictor of reading performance. However, the exact nature of the relationship between RAN performance and reading fluency is not clearly understood. Here we demonstrate how RAN performance may relate to early, mid-, and late-stage reading processes that can be measured using sophisticated variables provided by eye-tracking technology. Specifically, the current study employs eye tracking to measure essential aspects of reading fluency within early, mid, and late local (word) stages of the reading process and its associations with behavioral RAN.

To measure early local stages of the reading process, we used **first fixation duration** to reflect automatic, non-strategic reading or parsing procedures [60]. First fixation duration is defined as the duration of the first fixation made in an area of interest [57]. First fixation duration is considered one of the most important and highly reported eye-tracking measures and reflects orthographic processing/word recognition or lexical access [57,59,61].

Next, **regression path duration** is used as an intermediate (mid-stage) measure. Regression path duration is defined as the sum of fixations to previous words, after fixating on the target word [49]. Godfroid (2020) reports that regression path duration “is a more complex duration measure which considers the sequence of eye movements in addition to their duration” (p. 223) [57]. Regression path duration is a measure that is difficult to clearly classify as an early or late eye-tracking measure, as it taps into both early and late processes. As regression path duration combines characteristics of both processes, it is thus given an intermediary status [49] as the latest of the early measures or the earliest of the late measures. Regression path duration is indicative of difficulty integrating a word when it is fixated (early stage processing), while also reflecting the time to overcome lexical and integration difficulties (late-stage processing) [49,58,62,63,64]. Based on this understanding, heading further back in the text indicates a processing difficulty, while eye movements past the area of interest indicate the successful resolution of the difficulty that was encountered [57]. This may be a more meaningful measure for early readers as they are in a period of building reading automaticity and show greater variability in integrating different aspects of text. The interpretation of regression path duration can depend on the types of stimuli used. Typically, in syntactically rich sentences or paragraphs, regression path duration reflects higher-level integration or reanalysis difficulties and is frequently considered a late-stage measure. However, in this pilot study, as the stimuli consists of target words, regression path duration is categorized as a mid-local-stage measure.

Finally, total reading time is the most frequently reported eye-tracking measure represented across all text-based eye-tracking research. Total reading time is typically cited as a global measure of sentence processing. In the previous literature, total reading time has shown the strongest associations with learning [65]. As this study focuses on word reading, **total word reading time** is considered a late local measure. By carefully measuring early, mid, and late local aspects of word reading through the use of eye tracking, we can more objectively measure reading behaviors associated with fluent word reading as children move from being less skilled to more skilled readers.

In this study, we investigated the relationship between RAN performance and different stages of reading fluency captured by eye tracking in a sample of monolingual English-speaking first- and second-grade children, while accounting for age, word length, word frequency, and sentence condition. We used RAN (rapid letter naming (RLN) and rapid digit naming (RDN)) performance to predict first fixation duration as an early local measure, regression path duration as a mid-local measure, and total word reading time as a late-local measure of word reading fluency within a sentence-level reading task which was read aloud. By using eye-tracking variables as our dependent measures of reading fluency, we assess separate and unique aspects of early, mid, and late local reading behaviors to gain a better understanding of the nuances of fluency development in beginning readers. To the authors’ knowledge, this has not been performed before within the early school-aged population.

Further, RAN is a highly used clinical tool that assesses reading automaticity. RAN has been cited as a measure of lexical retrieval, yet the exact nature in which RAN relates to reading fluency remains unknown. By assessing the relationship between RAN and different stages of reading (i.e., early, mid, and late local measures), we can better determine the underlying cognitive processes associated with RAN performance. Given that our eye-tracking variables have been associated with different cognitive processes of reading (i.e., early stage = lexical retrieval; mid and late stage = integration of several phonological and orthographic processes), we can validate previous theoretical models regarding the underlying processes of RAN. To that end, we ask the following research questions:

1. Does RAN (RLN and RDN) predict the early local stage of word reading (first fixation duration) measured within a sentence-level eye-tracking task in first and second graders?
2. Does RAN (RLN and RDN) predict the mid-local stage of word reading (regression path duration) measured within a sentence-level eye-tracking task in first and second graders?
3. Does RAN (RLN and RDN) predict the late local stage of word reading (total word reading time) measured within a sentence-level eye-tracking task in first and second graders?

Hypothesis: We hypothesize that RAN performance will be related to all local stages of the word reading process, as it requires visual processing/recognition, lexical access, lexical retrieval (verbalization), and potentially reanalysis, all of which are involved in these stages.

## 2. Materials and Methods

### 2.1. Participants

The data for the current study were drawn from a larger cohort of 44 monolingual and Spanish–English bilingual students participating in a study on neurobiological markers of language and reading in early elementary school. The larger study included both EEG and eye-tracking tasks; however, only the eye-tracking task of the monolingual students will be discussed here. All students participated in behavioral language and literacy assessments and an eye-tracking reading task. Nine students were bilingual and excluded from these analyses as bilingualism could impact English reading development, e.g., [66]. Thirty-five monolingual (N = 27) and functionally monolingual (N = 8) students are the focus of this study, with an average age of 6.74 years (SD = 0.65). A functionally monolingual student is operationalized as one who has less than 20% use and exposure of another language and was unable to complete behavioral language testing in said language [67] (The *Bilingual Input Output Survey* (BIOS) [68] was administered to a parent of each student. All 8 students who were considered functionally monolingual had less than 10% use and exposure of Spanish based on hour-by-hour recounting. Group differences using an independent-samples *t*-test was conducted for all behavioral tests and there were no significant differences (*p* > 0.25); therefore, the monolingual and functionally monolingual groups were combined.) One participant was excluded due to inclusion in special education services and an additional participant was excluded due to the inability to complete the RAN task per standardized protocol. Therefore, a total of 33 students (12 females, 21 males (As gender identity was not a question that was asked on the questionnaire, biological sex is reported here)) in the first (N = 14) and second (N = 19) grades were included in the study. All parents and children gave informed consent/assent to participate in the study and were compensated for their participation. This study was approved by the Institutional Review Board at the University of Rhode Island as well as the elementary school’s administration team.

### 2.2. Behavioral Procedure

As part of the larger study, students participated in a comprehensive battery of behavioral assessments. To address the current research questions, the following tests were administered: the *Comprehensive Test of Phonological Processing—2nd Edition* (CTOPP-2) [69], the *Wechsler Abbreviated Scale of Intelligence—2nd Edition* (WASI-2) [70] and the *Clinical Evaluation of Language Fundamentals—5th Edition* (CELF-5) [71]. The WASI-2 and CELF-5 were used to confirm that all participants have nonverbal IQ scores and oral language that are within normal limits (standard scores above 85). Parents completed a language questionnaire [67] to gather information regarding demographics, including education and occupation statuses of both parents. The Hollingshead Four-Factor Index of Socioeconomic Status (SES) [72] was used to code parental education and parental occupation. Table 1 summarizes the demographic information for the participants in this study.

#### Comprehensive Test of Phonological Processing—2nd Edition (CTOPP-2)

The CTOPP-2, a standardized assessment that targets phonological awareness, phonological working memory, and rapid automatized naming, was used. The subtests administered included Elision, Blending Words, Sound Matching, Phoneme Isolation, Nonword Repetition, Memory for Digits, rapid digit naming (RDN), and rapid letter naming (RLN).

RLN and RDN subtests include four rows of nine letters/digits each, for a total of thirty-six items. The raw score is determined by the number of seconds needed to name all thirty-six items. The task is no longer considered automatic nor accurate when a participant makes four or more errors [73]; therefore, participants who had four or more errors were excluded from the analysis, resulting in the exclusion of 1 second-grade participant from further analyses. Although RLN and RDN can be analyzed with composite scores, e.g., [28], raw scores are more often used, e.g., [7,15,29,74,75], because they allow for more fine-grained analysis and do not group naming times. Therefore, RLN and RDN raw scores were used in this study. To ensure reliability of scores, a research assistant rescored the responses for all participants in the study. Interscorer reliability for both RLN and RDN was 100%.

### 2.3. Apparatus

Eye movements were recorded using an EyeLink Portable Duo (SR Research Ltd., Oakville, ON, Canada) with a sampling rate of 500 Hz. Stimuli sentences were presented on a 17.3-inch PC computer with a 40° visual angle. Stimuli sentences were presented in Times New Roman in black, size 20, on a white background. Participants did not use a chinrest during the task.

### 2.4. Eye-Tracking Task

The first author created a novel sentence-level reading task, which included 20 sentences with target words that are familiar to early school-age children (sample sentences are included in Appendix A). All but 3 of the target words (see Appendix B; Table A1) were taken from the Fry High-Frequency Word List (three target words were not on the Fry High-Frequency Word List but are still considered high-frequency words in the SubTLEX-US database. These words were selected by the first author to formulate sentences that were consistent with the text level while allowing for creation of a simple comprehension question) and all of the words were nouns [76]. The Fry list includes the most frequently occurring words in children’s text and these words are typically part of early school-age curricula. Two versions of the task were created as the larger study was expected to be longitudinal in nature (but was unfortunately disrupted due to COVID-19). It was our intention to counterbalance the task across years so that half of the participants received one version and half of the participants the other. The average log frequencies (log count words/million; SubTLEX-US, [77]) across versions did not differ (*p* = 0.46), suggesting that the text levels were equivalent.

One practice trial, with the target word “book” was followed by twenty sentences with embedded target words. Each target was embedded within either a short or long sentence. This manipulation was included as the previous literature has shown single fixation duration differences by sentence length and word position, i.e., [78]. All 20 targets appeared only once in each version of the experiment. Each version included ten short sentences (5–6 words) and ten long sentences (9–10 words) for a total of 20 sentences. The versions were balanced so that the target in version 1 was in a short sentence and in version 2, a long sentence (and vice versa). Half of the participants received one version and half of the participants the other. The eye-tracking variables (first fixation duration, regression path duration, and total word reading time) were measured on the target words within each sentence. Sentences were created to make certain that target words did not appear at the beginning and end of every sentence. Target words were not punctuated words, function words, proper nouns, repeated words, nor cross-linguistically ambiguous words, such as cognates and interlingual homographs, e.g., [79,80,81,82,83].

### 2.5. Eye-Tracking Procedure

The experiment was conducted in a windowless classroom. Participants underwent language and literacy testing during the school day. The eye-tracking task was completed in 1 session after school hours to ensure a quiet environment. The experiment was created using Experiment Builder software, version 1.10.165 [84]. A nine-point calibration and validation of the eye tracker was conducted for each participant prior to testing. Recalibration and validation were completed if there was a more than 2° *x*- or *y*-axis drift detected. Participants were instructed to read the sentences aloud and were audio recorded. Each trial was preceded by a drift check and fixation point, which triggered the presentation of the stimulus sentence. Each sentence was presented in a single line. Participants pressed any button to end a trial. Each trial was followed by a yes–no comprehension question presented auditorily through headphones to ensure that participants attended to the meaning of the sentences, e.g., [85]. Half of the comprehension questions had a correct response of yes, and half of no. The questions prompted overall processing of the entire sentence. Participants responded to the question by pressing the right (yes) or left (no) trigger button on a gamepad.

### 2.6. Data Cleaning Procedure

Frequently, data cleaning is not discussed within the methods section of peer-reviewed articles. Conklin and Pellicer-Sánchez (2016) explain the importance of including data cleaning procedure information for transparency and replicability [49]. The data cleaning was conducted in Data Viewer [86] and the data were cleaned in several stages. Trials were removed if the participant did not correctly read the target word aloud, resulting in the exclusion of 15.15% of trials. Then, the data were cleaned using a 3-stage cleaning method. Godfroid (2020) explains “that short fixations [50 to 100 ms] do not reflect cognitive processing… [and] it is better to merge or remove short fixations in applied research” (p. 261) [57]. Specifically, fixations of less than 80 ms and within 0.5° were merged with neighboring fixations, fixations of less than 40 ms and within 1.25° were merged with neighboring fixations, and any remaining fixations less than 80 ms were excluded from analysis [87]. The data were then drift-corrected to their average Y position to account for poor calibrations, participant movements, or drifts in gaze positions [57]. Drift corrections were first completed automatically and then manually, if necessary [57]. Manual drift corrections were required if one or more fixation position exceeded the batch drift correction threshold of 30. Any trials with less than three fixations were removed from analysis, which resulted in the exclusion of 7.29% of trials. Hessels and Hooge (2019) refer to “data loss” as track loss due to participant head turns, blinks, and/or the eye tracker’s “technical difficulties” (p. 2) [88]. Any trials with data loss were excluded, resulting in the exclusion of 2.43% of trials. Details of all data loss were consolidated into a table provided in the Supplementary Materials.

### 2.7. Eye-Tracking Reading Measures

Three eye-tracking measures were used to capture early (i.e., first fixation duration), mid (i.e., regression path duration), and late (i.e., total word reading time) local reading processes used for reading of the target words embedded within each sentence. First fixation is the duration of the first fixation on a target region. Regression path duration is the sum of fixations to earlier parts of the sentence, after fixating on the target word. Therefore, only regressions to previous words were coded and analyzed (within-word regressions were not included in this measure). Total word reading time is defined as the total sum of all fixation durations recorded for a target region. Each variable is analyzed individually with RLN and RDN raw scores.

### 2.8. Statistical Analyses

Preliminary checks and correlations of the data were completed to assess normality of distributions. Item-level analysis was conducted for the three eye-tracking measures. Any item that was more than 3 SDs from the mean (an outlier), was excluded from further analyses (2.10% of all eye-tracking items). For smaller datasets, a linear mixed-effects model (LMEM) with log-transformed dependent variables is often preferred, as it improves normality and homoscedasticity of residuals without the complexity and instability that can arise from fitting generalized linear mixed models [83,84]. First, the correlation between RLN and RDN was calculated and showed that they are highly correlated (r(33) = 0.79, *p* < 0.001). The means and standard deviations for the three eye-tracking variables are shown in Table 2. Additionally, the eye-tracking variables are moderately correlated with one another (Table 3).

To address each research question, linear mixed-effects models (LMEMs) were conducted with the eye-tracking measures as the outcome variables. To control for age, sentence condition (short or long sentence length), target word length, target word frequency, and target word position, these variables were included in each linear mixed-effects model. Age was included given that the dependent variables were unstandardized.

## 3. Results

To investigate Research Question 1, first fixation duration and RAN variables were analyzed using an LMEM. The RLN model did not significantly predict first fixation duration while controlling for word frequency, word length, word position, sentence condition, and age (*p* > 0.05; see Table 4 for full results). However, age was a significant predictor of first fixation duration when RDN was included in the model (β = 0.12, t = 2.08, *p* = 0.04; see Table 5 for full results). Older participants tended to have a longer first fixation duration, controlling for word frequency, word length, word position, and sentence condition.

To investigate Research Question 2, regression path duration and RAN variables were analyzed. RLN was a significant predictor of regression path duration, with a positive effect (β = 0.02, t = 2.48, *p* = 0.02; see Table 6 for full results). Participants with longer RLN times tended to have longer regression path durations, controlling for word frequency, word length, sentence condition, and age. RDN was also a significant predictor of regression path duration, with a positive effect (β = 0.02, t = 2.26, *p* = 0.03; see Table 7 for full results).

To investigate Research Question 3, total word reading time and RAN variables were analyzed. Neither RLN nor RDN significantly predicted total word reading time while controlling for word frequency, word length, word position, sentence condition, and age (*p* > 0.05; see Table 8 and Table 9 for full results). (Post hoc models were analyzed with both phonological awareness (the phonological awareness composite score on the CTOPP) and receptive vocabulary (ROWPVT) in each LMEM. These analyses found no difference in results for first fixation duration, regression path duration, and total duration for LMEMs with RLN. Likewise, for the LMEMs with RDN, phonological awareness and vocabulary were not significant predictors of our eye-tracking measures in any of the models).

As RLN and RDN are highly correlated in a post hoc analysis, a principal components analysis was conducted on these two variables to extract their shared variance. The first principal component accounted for 86% of the variance and loaded strongly on both RLN and RDN. This component was used as a single predictor in the LME models below (see Table 10, Table 11 and Table 12).

## 4. Discussion

The purpose of this pilot study was to determine the relationship between rapid automatized naming (RAN) and early, mid, and late local stages of the reading process within a novel eye-tracking task. RAN is a highly valid and reliable clinical tool that is predictive of word-reading skill for young children, yet what remains unknown is the nature in which RAN is associated with different aspects of word reading fluency. The use of eye tracking allowed for the investigation of subtle, yet distinct aspects of early, mid, and late local stages of reading fluency. By using eye tracking to measure these stages, we have the potential to elucidate nuances in word reading behaviors (e.g., fixation durations, more automatic word recognition vs. reanalysis/integration, or deeper lexical processing, etc.) that cannot be captured directly by traditional behavioral measures alone (e.g., reading accuracy and reading time).

We first asked whether RAN variables, RLN and RDN, predicted first fixation duration as a measure of early reading processes. First fixation duration reflected the duration of the first fixation in a target region. This variable is considered a process that occurs in the initial stages of sentence processing [58] and reflects orthographic processing/word recognition or lexical access [57,59,61]. RLN and RDN performance did not predict first fixation duration. There are several possible reasons for this result. One, the target words used within this study may not have been orthographically complex enough to elicit longer looking times at the target word. Second, first fixation duration as an early stage reading process may not be as highly associated with RAN performance compared to mid and late local stages of the reading process, as it may be more associated with simple visual scanning and non-strategic reading or parsing procedures see [60,89]. First fixation duration may only tap lexical access processes but does not necessarily reflect the time in which articulatory processes are activated to produce a word. Lastly, given the serial nature of our RAN task, performance may necessitate integrative processes, including self- monitoring strategies, which are more associated with mid- and late-stage variables.

Next, we asked whether RAN variables, RLN and RDN, predicted the mid-stage of word reading in first- and second-grade readers. In this pilot study, regression path duration was used as an index of mid-stage word reading. As previously mentioned, in previous studies with syntactically rich text, regression path duration reflects higher-level integration or reanalysis difficulties (thus categorized as alate-stage measure). As a mid-local stage measure, regression path duration may reflect local word recognition or phonological processing difficulties. Regression path duration may also be related to higher-level processing as the reader attempts to comprehend and integrate what they had recently read prior to the target word. The regression path duration variable measures the sum of fixations to earlier parts of the sentence, after fixating on the target word. Increased RLN and RDN times were associated with a longer regression path duration. This is significant as it may reflect behaviors associated with less automatic reading. It also links poor RAN performance with weaknesses in the integration of several linguistic processes necessary for text comprehension.

Finally, we asked whether RAN variables predicted a late local measure of reading, total word reading time. Total word reading time allowed for measurement of the sum of all fixation durations recorded for a target region. In this case, the target region was a word embedded within a sentence. Neither RLN nor RDN significantly predicted total reading time. Total reading time, the most frequently reported eye-tracking measure in text-based research, is generally considered a global indicator of sentence processing. In the present study, however, it was applied as a *local* measure, calculated for individual word stimuli rather than at the sentence level. This word-level approach may have reduced its sensitivity to integrative processes, making it a less effective index of global late-stage reading skills. In contrast, regression path duration captures regressions to prior words, thereby reflecting mid-stage integrative processing. Overall, because RLN and RDN were associated with measures indexing integration, behavioral RAN performance may primarily reflect integrative rather than early stage lexical access processes. This finding has important theoretical implications, as RAN has traditionally been viewed as a proxy for lexical access. Future eye-tracking studies are needed to clarify how RAN relates to the oculomotor, cognitive, and linguistic mechanisms underlying fluent reading.

In a post hoc analysis, as RLN and RDN were highly correlated, a principal components analysis was conducted on these two variables to extract their shared variance. This component was used as a single predictor in the LME models. As noted similarly above, the component that included the shared variance between RLN and RDN did not predict first fixation duration, did predict regression path duration, and did not predict total reading time.

### 4.1. Clinical Implications

Assessment practices are particularly important to clinicians serving early school-aged children. Busy clinicians require assessment tools that are time-efficient and still diagnostically sensitive. School-based clinicians are often serving large caseloads and may have limited time for testing. Also, young children often tire from extensive testing batteries; therefore, choosing assessment tools that are quick to administer, yet allow for sensitive measurement, is paramount. The preliminary results of this study support the notion that RLN and RDN are tightly linked and therefore perhaps we can use one of these measures to streamline testing. Given the fact that children with DLD, RD, and comorbid DLD and RD may have a distinct deficit in RLN skill, RDN may not need to be included in clinical assessments for young readers [5,19,75]. Perhaps RLN alone may be a sensitive indicator of reading fluency that can be used within a comprehensive diagnostic battery for young children [16]. Future studies with larger sample sizes can consider if it is possible to shorten a test battery to include only the most sensitive RAN task(s).

Our preliminary results also demonstrate that children who exhibit increased times in RLN and RDN tasks may show difficulties in reading automaticity and may require further time or several attempts at rereading to more fully extract meaning from text. Therefore, early school-aged children demonstrating below-average performance on RAN tasks may benefit from evidence-based interventions that increase reading automaticity (i.e., repeated reading, direct orthographic instruction, explicit vocabulary instruction, peer-mediated strategies, and active self-monitoring; [90]). Fluent word reading is essential to the general reading process as it allows cognitive resources to focus on the comprehension of text, which is the end goal of the reading process.

### 4.2. Limitations

These preliminary findings should be interpreted in the context of a few limitations. This sample size included 33 monolingual and functionally monolingual English-speaking children. A formal power analysis suggests that approximately 70 participants would be needed to detect moderate effects with 80% power at an alpha level of 0.05. Therefore, the present study may have been underpowered to detect small-to-moderate associations between RDN and first fixation duration and RDN (*p* = 0.13), as well as between RLN and total word reading time (*p* = 0.07). These null findings should be interpreted with caution, as such effects may be detectable with a larger sample size. Future studies with larger sample sizes and greater language diversity (i.e., multilingual learners) are needed so that results may be more widely generalized. Further, the sample contained children with typical language. Therefore, the relationship between RAN and measures of early, mid, and late local stages of reading fluency in the context of children with language disorders cannot be determined at this time. Additionally, dyslexia was not screened and thus some of these children with typical oral language may still have reading difficulties. It is possible that clinical populations may show different patterns in these processes or may be affected to a greater degree.

We acknowledge that eye-tracking procedures are more complicated in young children who are prone to movement or may have difficulties sitting still for extended periods of time. Therefore, data loss is common due to artifacts; however, great lengths were taken to establish a systematic and high-quality data cleaning procedure to ensure the validity of the experimental data. Lastly, this is the first step in establishing a relationship between RAN performance and early, mid, and late eye-tracking variables in early school-aged children. As suggested by our hypothesis, we predicted that there would be an association between RAN performance and an early stage eye-tracking variable (i.e., first fixation duration) given that first fixation duration has been reported as a measure of lexical access. Our analyses demonstrated a relationship between the mid-stage of reading fluency only, suggesting that RAN performance was associated with more integrative processes. Further investigations are warranted to determine how first fixation duration, regression path duration, and total word reading time measures capture different aspects of the reading process and how these variables are related to RAN performance in early readers.

## 5. Conclusions

This pilot study provides further evidence that RAN is a predictor of word reading fluency in early school-aged children. Here is the first study known to these authors that quantifies aspects of **early** (first fixation duration), **mid** (regression path duration), and **late** (total reading time) local reading processes through the use of eye-tracking technology in English-speaking early school-aged children. Behavioral RAN, specifically RLN and RDN, predicted mid-stage word reading fluency processes as indexed by regression path duration. Children with slower RAN times exhibited longer regressions during reading, potentially reflecting challenges in the rapid *integration* of visual and phonological information.

## Figures and Tables

**Table 1:** Participant descriptive statistics in means (M) and standard deviations (SD).

Measure	M	SD	Range
Age (in months)	85.50	8.04	74–103
Parent 1 Education ^a^	5.66	1.01	3–7
Parent 2 Education ^a^	4.62	2.43	0–7
Parent 1 Occupation ^b^	5.38	2.55	1–9
Parent 2 Occupation ^b^	4.03	3.01	0–9
RLN Raw Score (in seconds)	26.16	7.23	18–45
RDN Raw Score (in seconds)	23.22	5.44	14–38
RAN Composite Score ^c^	102.25	7.99	88–119
CELF-5 Core Language Score ^d^	101.5	9.70	80–120
WASI-2 Perceptual Reasoning Composite Score ^d^	101.28	12.04	75–128

RLN = rapid letter naming; RDN = rapid digit naming; RAN = rapid automatized naming; CELF-5 = Clinical Evaluation of Language Fundamentals—5th Edition; WASI = Wechsler Abbreviated Scale Intelligence—2nd Edition. ^a^ Hollingshead Index, parental education was measured using the following scale: 1 = less than 7th grade, 2 = junior high school, including 9th grade, 3 = partial high school, 10th or 11th grade, 4 = high school graduate, 5 = partial college or at least one year of specialized training, 6 = standard college or university graduation, and 7 = graduate or professional training. ^b^ Hollingshead Index, parental occupation was measured using the following scale: 0 = not applicable or unknown, students, housewives, unemployed; 1 = farm laborers, menial service workers, students, housewives (dependent on welfare, no regular occupation); 2 = unskilled workers; 3 = machine operators and semiskilled workers; 4 = smaller business owners (<USD 25,000), skilled manual laborers, craftsmen, tenant farmers; 5 = clerical and sales workers, small farm and business owners (business valued at USD 25,000–USD 50,000); 6 = technicians, semiprofessionals, small business owners (business valued at USD 50,000–USD 70,000); 7 = smaller business owners, farm owners, managers, minor professionals; 8 = administrators, lesser professionals, proprietor of medium-sized business; and 9 = higher executive, proprietor of large businesses, major professional. ^c^ This is a standardized composite score of RLN and RDN. ^d^ This is a standardized score.

**Table 2:** Eye-tracking measures in means (M) and standard deviations (SD).

Measure	M	SD	Range
First Fixation Duration (in msec)	380.60	181.86	81–799
Regression Path Duration (in msec)	611.20	399.76	128–2192
Total Word Reading Time (in msec)	616.53	352.85	125–1827

**Table 3:** Correlations between eye-tracking measures.

Measure	Regression Path Duration	Total Word Reading Time
First Fixation Duration	0.28 ***	0.39 ***
Regression Path Duration		0.54 ***

Note. *** *p*-value < 0.001.

**Table 4:** Linear mixed-effects model for first fixation duration and RLN.

Predictor	Estimate	Standard Error	df	*t*-Value	*p*-Value
**Intercept**	**4.33**	**0.68**	**85.87**	**6.47**	**<0.001**
RLN	0.004	0.01	35.14	0.65	0.52
Word Frequency	0.22	0.11	258.34	1.94	0.05
Word Length	−0.02	0.03	259.62	−0.75	0.45
Word Position	−0.01	0.02	261.74	−0.63	0.53
Sentence Condition	0.02	0.02	258.68	1.24	0.22
Age	0.09	0.06	31.73	1.62	0.11
Random Effects					
Group Participant (intercept)	Variance	SD			
	0.002	0.04			
Residual	0.26	0.51			

Note. Significant results are indicated in bold.

**Table 5:** Linear mixed-effects model for first fixation duration and RDN.

Predictor	Estimate	Standard Error	df	*t*-Value	*p*-Value
**Intercept**	**3.97**	**0.70**	**263.00**	**5.65**	**<0.001**
RLN	0.01	0.01	263.00	1.52	0.13
Word Frequency	0.22	0.11	263.00	1.95	0.05
Word Length	−0.02	0.03	263.00	−0.74	0.46
Word Position	−0.01	0.02	263.00	−0.49	0.62
Sentence Condition	0.02	0.02	263.00	1.15	0.25
**Age**	**0.12**	**0.06**	**263.00**	**2.08**	**0** **.04**
Random Effects					
Group Participant (intercept)	Variance	SD			
	0.00	0.00			
Residual	0.26	0.51			

Note. Significant results are indicated in bold.

**Table 6:** Linear mixed-effects model for regression path duration and RLN.

Predictor	Estimate	Standard Error	df	*t*-Value	*p*-Value
**Intercept**	**4.97**	**0.90**	**65.00**	**5.50**	**<0.001**
**RLN**	**0.02**	**0.01**	**29.85**	**2.48**	**0** **.02**
Word Frequency	0.04	0.14	241.02	0.28	0.78
Word Length	0.01	0.03	241.88	0.37	0.71
Word Position	0.03	0.03	245.65	1.17	0.25
Sentence Condition	−0.01	0.02	241.26	−0.60	0.55
Age	0.08	0.08	24.76	1.00	0.33
Random Effects					
Group Participant (intercept)	Variance	SD			
	0.01	0.12			
Residual	0.37	0.61			

Note. Significant results are indicated in bold.

**Table 7:** Linear mixed-effects model for regression path duration and RDN.

Predictor	Estimate	Standard Error	df	*t*-Value	*p*-Value
**Intercept**	**4.86**	**0.96**	**57.71**	**5.06**	**<0.001**
**RDN**	**0.02**	**0.01**	**22.96**	**2.26**	**0.03**
Word Frequency	0.04	0.14	240.50	0.29	0.77
Word Length	0.01	0.03	242.08	0.28	0.78
Word Position	0.03	0.03	242.83	1.30	0.19
Sentence Condition	−0.02	0.02	239.49	−0.73	0.46
Age	0.09	0.08	26.15	1.10	0.28
Random Effects					
Group Participant (intercept)	Variance	SD			
	0.02	0.12			
Residual	0.37	0.61			

Note. Significant results are indicated in bold.

**Table 8:** Linear mixed-effects model for total word reading and RLN.

Predictor	Estimate	Standard Error	df	*t*-Value	*p*-Value
**Intercept**	**4.66**	**0.82**	**71.40**	**5.70**	**<0.001**
RLN	0.01	0.01	20.49	1.87	0.07
Word Frequency	0.18	0.13	253.42	1.34	0.18
Word Length	0.03	0.03	254.68	0.94	0.35
Word Position	0.003	0.03	257.38	0.13	0.89
Sentence Condition	−0.02	0.02	253.74	−0.89	0.38
Age	0.10	0.07	25.76	1.45	0.16
Random Effects					
Group Participant (intercept)	Variance	SD			
	0.004	0.06			
Residual	0.36	0.60			

Note. Significant results are indicated in bold.

**Table 9:** Linear mixed-effects model for total word reading and RDN.

Predictor	Estimate	Standard Error	df	*t*-Value	*p*-Value
**Intercept**	**4.74**	**0.88**	**62.55**	**5.37**	**<0.001**
RDN	0.01	0.01	23.36	1.33	0.20
Word Frequency	0.18	0.13	252.47	1.33	0.19
Word Length	0.03	0.03	254.47	0.84	0.40
Word Position	0.01	0.03	254.34	0.20	0.84
Sentence Condition	−0.02	0.02	251.10	−0.95	0.34
Age	0.10	0.07	27.76	1.28	0.21
Random Effects					
Group Participant (intercept)	Variance	SD			
	0.01	0.08			
Residual	0.36	0.60			

Note. Significant results are indicated in bold.

**Table 10:** Linear mixed-effects model for first fixation duration and shared variance of RLN and RDN (SV RLN and RDN).

Predictor	Estimate	Standard Error	df	*t*-Value	*p*-Value
**Intercept**	**4.28**	**0.63**	**106.49**	**6.77**	**<0.001**
SV RLN and RDN	−0.03	0.03	30.77	−1.21	0.24
Word Frequency	0.22	0.11	258.75	1.95	0.05
Word Length	−0.02	0.03	260.42	−0.72	0.47
Word Position	−0.01	0.02	261.72	−0.57	0.57
Sentence Condition	0.02	0.02	258.79	1.21	0.23
Age	0.11	0.06	32.07	1.91	0.07
Random Effects					
Group Participant (intercept)	Variance	SD			
	0.0001	0.01			
Residual	0.26	0.51			

Note. Significant results are indicated in bold.

**Table 11:** Linear mixed-effects model for regression path duration and shared variance of RLN and RDN (SV RLN and RDN).

Predictor	Estimate	Standard Error	df	*t*-Value	*p*-Value
**Intercept**	**5.25**	**0.83**	**81.30**	**6.32**	**<0.001**
**SV RLN and RDN**	**−0.11**	**0.04**	**26.14**	**−2.76**	**0.01**
Word Frequency	0.04	0.14	241.38	0.28	0.78
Word Length	0.01	0.03	242.82	0.37	0.71
Word Position	0.03	0.03	245.35	1.26	0.21
Sentence Condition	−0.02	0.02	241.41	−0.66	0.51
Age	0.11	0.08	24.36	1.34	0.19
Random Effects					
Group Participant (intercept)	Variance	SD			
	0.01	0.11			
Residual	0.38	0.61			

Note. Significant results are indicated in bold.

**Table 12:** Linear mixed-effects model for total word reading and shared variance of RLN and RDN (SV RLN and RDN).

Predictor	Estimate	Standard Error	df	*t*-Value	*p*-Value
**Intercept**	**4.87**	**0.77**	**85.58**	**6.35**	**<0.001**
SV RLN and RDN	−0.06	0.03	25.08	−1.88	0.07
Word Frequency	0.18	0.13	253.23	1.34	0.18
Word Length	0.03	0.03	254.96	0.91	0.36
Word Position	0.01	0.03	256.61	0.21	0.83
Sentence Condition	−0.02	0.02	252.89	−0.95	0.35
Age	0.11	0.07	24.88	1.58	0.13
Random Effects					
Group Participant (intercept)	Variance	SD			
	0.003	0.05			
Residual	0.36	0.60			

Note. Significant results are indicated in bold.

## Data Availability

The raw data supporting the conclusions of this article will be made available by the authors on request.

## Supplementary Materials

The following supporting information can be downloaded at: https://www.mdpi.com/article/10.3390/jemr19010016/s1, Supplementary Table S1: Data Loss by Exclusionary Criteria.

## Abbreviations

The following abbreviations are used in this manuscript:

RAN	Rapid Automatized Naming
RLN	Rapid Letter Naming
RDN	Rapid Digit Naming
RD	Reading Disability
DLD	Developmental Language Disorder
BIOS	Bilingual Input Output Survey
CTOPP-2	Comprehensive Test of Phonological Processing—2nd Edition
WASI-2	Weschler Abbreviated Scale of Intelligence—2nd Edition
CELF-5	Clinical Evaluation of Language Fundamentals—5th Edition
LMEM	Linear Mixed-Effects Model

## Appendix A

Sample sentences:
We took a plane to grandma’s.
The pilot of the plane went over the safety rules.

I slipped on ice shoveling snow.
Yesterday, the ice was too thin to skate on.

The farmer rode his tractor.
The farmer let me taste some of his vegetables.

## Appendix B

**Table A1:** List 1 and 2 target words.

List 1	List 2
king	bed
cow	bell
rain	ice
sea	paint
window	sun
space	oil
tree	mouth
umbrella	apple
farm	soil
cotton	wave
dog	mountain
horse	bear
cloud	farmer
lake	factory
corn	clock
fish	bird
ear	yard
cat	hat
plane	milk
nose	grass

## References

[1] Hogan T.P., Catts H.W., Little T.D. (2005). The relationship between phonological awareness and reading. Lang. Speech Hear. Serv. Sch..

[2] Wagner R.K., Torgensen J.K. (1987). The nature of phonological processing and its role in the acquisition of reading skills. Psychol. Bull..

[3] Denckla M.B., Rudel R. (1974). Rapid “automatized” naming of pictured objects, colors, letters and numbers by normal children. Cortex.

[4] Wagner R.K., Torgesen J.K., Rashotte C.A., Hecht S.A., Barker T.A., Burgess S.R., Donahue J., Garon T. (1997). Changing relations between phonological processing abilities and word-level reading as children develop from beginning to skilled readers: A 5-year longitudinal study. Dev. Psychol..

[5] Norton E.S., Wolf M. (2012). Rapid automatized naming (RAN) and reading fluency: Implications for understanding and treatment of reading disabilities. Annu. Rev. Psychol..

[6] de Jong P.F. (2011). What discrete and serial rapid automatized naming can reveal about reading. Sci. Stud. Read..

[7] Lervåg A., Hulme C. (2009). Rapid automatized naming (RAN) taps a mechanism that places constraints on the development of early reading fluency. Psychol. Sci..

[8] Vellutino F.R., Fletcher J.M., Snowling M.J., Scanlon D.M. (2004). Specific reading disability (dyslexia): What have we learned in the past four decades?. J. Child Psychol. Psychiatry.

[9] Wimmer H., Mayringer H., Landerl K. (2000). The double-deficit hypothesis and difficulties in learning to read a regular orthography. J. Educ. Psychol..

[10] Gray J.S., Powell-Smith K.A. (2025). Rapid automatized naming: What it is, what it is not, and why it matters. Ann. Dyslexia.

[11] Wolf M., Bowers P.G. (1999). The double-deficit hypothesis for the developmental dyslexias. J. Educ. Psychol..

[12] Al Dahhan N.Z., Kirby J.R., Brien D.C., Gupta R., Harrison A., Munoz D.P. (2020). Understanding the biological basis of dyslexia at a neural systems level. Brain Commun..

[13] Al Dahhan N.Z., Kirby J.R., Chen Y., Brien D.C., Munoz D.P. (2020). Examining the neural and cognitive processes that underlie reading through naming speed tasks. Eur. J. Neurosci..

[14] Peters J.L., Bavin E.L., Crewther S.G. (2020). Eye movements during RAN as an operationalization of the RAN-reading “microcosm”. Front. Hum. Neurosci..

[15] Kirby J.R., Parrila R.K., Pfeiffer S.L. (2003). Naming speed and phonological awareness as predictors of reading development. J. Educ. Psychol..

[16] Gordon P.C., Hoedemaker R.S. (2016). Effective scheduling of looking and talking during rapid automatized naming. J. Exp. Psychol. Hum. Percept. Perform..

[17] Wolff U. (2014). RAN as a predictor of reading skills, and vice versa: Results from a randomised reading intervention. Ann. Dyslexia.

[18] McWeeny S., Choi S., Choe J., LaTourrette A., Roberts M.Y., Norton E.S. (2022). Rapid automatized naming (RAN) as a kindergarten predictor of future reading in English: A systematic review and meta-analysis. Read. Res. Q..

[19] De Groot B.J., Van den Bos K.P., Van der Meulen B.F., Minnaert A.E. (2015). Rapid naming and phonemic awareness in children with reading disabilities and/or specific language impairment: Differentiating processes?. J. Speech Lang. Hear. Res..

[20] Parrila R., Kirby J.R., McQuarrie L. (2004). Articulation rate, naming speed, verbal short-term memory, and phonological awareness: Longitudinal predictors of early reading development?. Sci. Stud. Read..

[21] Vaessen A., Blomert L. (2010). Long-term cognitive dynamics of fluent reading development. J. Exp. Child Psychol..

[22] Haager D., Dimino J., Windmueller M. (2014). Interventions for Reading Success.

[23] Kim Y.-S.G., Quinn J.M., Petscher Y. (2021). What is text reading fluency and is it a predictor or an outcome of reading comprehension? A longitudinal investigation. Dev. Psychol..

[24] Protopapas A., Katopodi K., Altani A., Kolotoura I., Ziaka L., Georgiou G.K. (2024). A process-oriented analysis of speech and silent intervals in responses to serial naming tasks. J. Educ. Psychol..

[25] Araújo S., Faísca L. (2019). A meta-analytic review of naming-speed deficits in developmental dyslexia. Sci. Stud. Read..

[26] Georgiou G.K., Parrila R., Cui Y., Papadopoulos T.C. (2013). Why is rapid automatized naming related to reading?. J. Exp. Child Psychol..

[27] Logan J.A.R., Schatschneider C., Wagner R.K. (2011). Rapid serial naming and reading ability: The role of lexical access. Read. Writ..

[28] Wood C., Bustamante K., Fitton L., Brown D., Petscher Y. (2017). Rapid automatic naming performance of young Spanish–English speaking children. Languages.

[29] Araújo S., Reis A., Petersson K.M., Faísca L. (2015). Rapid automatized naming and reading performance: A meta-analysis. J. Educ. Psychol..

[30] Bowey J.A., McGuigan M., Ruschena A. (2005). On the association between serial naming speed for letters and digits and word-reading skill: Towards a developmental account. J. Res. Read..

[31] Savage R., Frederickson N. (2005). Evidence of a highly specific relationship between rapid automatic naming of digits and text-reading speed. Brain Lang..

[32] Cronin V., Carver P. (1998). Phonological sensitivity, rapid naming, and beginning reading. Appl. Psycholinguist..

[33] Cummine J., Szepesvari E., Chouinard B., Hanif W., Georgiou G.K. (2014). A functional investigation of RAN letters, digits, and objects: How similar are they?. Behav. Brain Res..

[34] Di Filippo G., Brizzolara D., Chilosi A., De Luca M., Judica A., Pecini C., Spinelli D., Zoccolotti P. (2005). Rapid naming, not cancellation speed or articulation rate, predicts reading in an orthographically regular language (Italian). Child Neuropsychol..

[35] Georgiou G., Stewart B. (2013). Is rapid automatized naming automatic?. Presch. Prim. Educ..

[36] Mazzocco M.M., Grimm K.J. (2013). Growth in rapid automatized naming from grades K to 8 in children with math or reading disabilities. J. Learn. Disabil..

[37] Närhi V., Ahonen T., Aro M., Leppäsaari T., Korhonen T.T., Tolvanen A., Lyytinen H. (2005). Rapid serial naming: Relations between different stimuli and neuropsychological factors. Brain Lang..

[38] Van den Bos K.P., Zijlstra B.J.H., Lutje Spelberg H.C. (2002). Life-span data on continuous-naming speeds of numbers, letters, colors, and pictured objects, and word-reading speed. Sci. Stud. Read..

[39] Georgiou G.K., Parrila R., Manolitsis G., Kirby J.R. (2011). Examining the Importance of Assessing Rapid Automatized Naming (RAN) for the Identification of Children with Reading Difficulties. Learn. Disabil. A Contemp. J..

[40] Hornung C., Martin R., Fayol M. (2017). General and specific contributions of RAN to reading and arithmetic fluency in first graders: A longitudinal latent variable approach. Front. Psychol..

[41] Bishop D.V.M., Snowling M.J. (2004). Developmental dyslexia and specific language impairment: Same or different?. Psychol. Bull..

[42] Bignon M., Mejias S., Casalis S. (2025). Cognitive predictors of decoding skills in newcomer and monolingual French-speaking children. J. Speech Lang. Hear. Res..

[43] Bonifacci P., Cangelosi M., Bellocchi S. (2024). Oral language predictors of word reading and spelling: A cross-linguistic comparison in bilingual and monolingual children. J. Exp. Child Psychol..

[44] Kishchak V., Ewert A., Halczak P., Kleka P., Szczerbiński M. (2024). RAN and two languages: A meta-analysis of the RAN-reading relationship in bilingual children. Read. Writ..

[45] Papadopoulos T.C., Spanoudis G.C., Georgiou G.K. (2016). How is RAN related to reading fluency? A comprehensive examination of the prominent theoretical accounts. Front. Psychol..

[46] Caravolas M., Lervåg A., Defior S., Málkoá G.S., Hulme C. (2013). Different patterns, but equivalent predictors, of growth in reading in consistent and inconsistent orthographies. Psychol. Sci..

[47] Georgiou G.K., Papadopoulos T.C., Kaizer E.L. (2014). Different RAN components relate to reading at different points in time. Read. Writ..

[48] Puolakanaho A., Ahonen T., Aro M., Eklund K., Leppänen P.H., Poikkeus A., Tolvanen A., Torppa M., Lyytinen H. (2007). Very early phonological and language skills: Estimating individual risk of reading disability. J. Child Psychol. Psychiatry.

[49] Conklin K., Pellicer-Sánchez A. (2016). Using eye-tracking in applied linguistics and second language research. Second Lang. Res..

[50] Easson K., Al Dahhan N.Z., Brien D.C., Kirby J.R., Munoz D.P. (2020). Developmental trends of visual processing of letters and objects using naming speed tasks. Front. Hum. Neurosci..

[51] Christoforou C., Fella A., Leppänen P.H., Georgiou G.K., Papadopoulos T.C. (2021). Fixation-related potentials in naming speed: A combined EEG and eye-tracking study on children with dyslexia. Clin. Neurophysiol..

[52] Al Dahhan N.Z., Kirby J.R., Brien D.C., Munoz D.P. (2017). Eye movements and articulations during a letter naming speed task: Children with and without dyslexia. J. Learn. Disabil..

[53] Al Dahhan N.Z., Kirby J.R., Munoz D.P. (2016). Understanding reading and reading difficulties through naming speed tasks: Bridging the gaps among neuroscience, cognition, and education. AERA Open.

[54] Pan J., Yan M., Laubrock J., Shu H., Kliegl R. (2013). Eye-voice span during rapid automatized naming of digits and dice in Chinese normal and dyslexic children. Dev. Sci..

[55] Breznitz Z. (2003). Speed of phonological and orthographic processing as factors in dyslexia: Electrophysiological evidence. Genet. Soc. Gen. Psychol. Monogr..

[56] Godfroid A., Hui B. (2020). Five common pitfalls in eye-tracking research. Second Lang. Res..

[57] Godfroid A. (2020). Eye Tracking in Second Language Acquisition and Bilingualism: A Research Synthesis and Methodological Guide.

[58] Clifton C., Staub A., Rayner K. (2007). Eye movements in reading words and sentences. Eye Movements.

[59] Kim Y., Petscher Y., Vorstius C. (2020). The relations of online reading processes (eye movements) with working memory, emergent literacy skills, and reading proficiency. Sci. Stud. Read..

[60] Godfroid A., Winke P., Rebuschat P. (2015). Investigating implicit and explicit processing using L2 learners’ eye-movement data. Implicit and Explicit Learning of Languages.

[61] Perry C., Ziegler J.C., Zorzi M. (2010). Beyond single syllables: Large-scale modeling of reading aloud with the Connectionist Dual Process (CDP++) model. Cogn. Psychol..

[62] Radach R., Kennedy A. (2004). Theoretical perspectives on eye movements in reading: Past controversies, current issues, and an agenda for future research. Eur. J. Cogn. Psychol..

[63] Rayner K., Pollatsek A. (2006). Eye-movement control in reading. Handbook of Psycholinguistics.

[64] Reilly R.G., Radach R. (2006). Some empirical tests of an interactive activation model of eye movement control in reading. Cogn. Syst. Res..

[65] Godfroid A., Ahn J., Choi I., Ballard L., Cui Y., Johnston S., Lee S., Sarkar A., Yoon H.-J. (2018). Incidental vocabulary learning in a natural reading context: An eye-tracking study. Biling. Lang. Cogn..

[66] Bialystok E., Bhatia T.K., Ritchie W.C. (2012). The impact of bilingualism on language and literacy development. The Handbook of Bilingualism and Multilingualism.

[67] Bedore L., Fiestas C., Peña E., Nagy V. (2006). Cross-language comparisons of maze use in Spanish and English in functionally monolingual and bilingual children. Biling. Lang. Cogn..

[68] Peña E.D., Gutiérrez-Clellen V.F., Iglesias A., Goldstein B.A., Bedore L.M. (2018). Bilingual Input/Output Survey (BIOS).

[69] Wagner R., Torgesen J., Rashotte C., Pearson N. (2013). CTOPP: Comprehensive Test of Phonological Processing—Second Edition.

[70] Wechsler D. (2011). Wechsler Abbreviated Scale of Intelligence—Second Edition (WASI-II).

[71] Wiig E.H., Semel E., Secord W.A. (2013). Clinical Evaluation of Language Fundamentals—Fifth Edition (CELF-5).

[72] Hollingshead A.A. (1975). Four-Factor Index of Social Status.

[73] Norton E. (2019). Linking Rapid Automatized Naming (RAN), Reading, and the Brain.

[74] Liu C., Georgiou G.K. (2017). Cognitive and environmental correlates of rapid automatized naming in Chinese kindergarten children. J. Educ. Psychol..

[75] Ozernov-Palchik O., Norton E.S., Sideridis G., Beach S.D., Wolf M., Gabrieli J.D.E., Gaab N. (2017). Longitudinal stability of pre-reading skill profiles of kindergarten children: Implications for early screening and theories of reading. Dev. Sci..

[76] Fry E. (1980). The new instant word list. Read. Teach..

[77] Brysbaert M., New B. (2009). Moving beyond Kucera and Francis: A critical evaluation of current word frequency norms and the introduction of a new and improved word frequency measure for American English. Behav. Res. Methods.

[78] Kuperman V., Dambacher M., Nuthmann A., Kliegl R. (2010). The effect of word position on eye-movements in sentence and paragraph reading. Q. J. Exp. Psychol..

[79] Miellet S., Sparrow L., Sereno S.C. (2007). Word frequency and predictability effects in reading French: An evaluation of the EZ Reader model. Psychon. Bull. Rev..

[80] Whitford V., Titone D. (2012). Second-language experience modulates first- and second-language word frequency effects: Evidence from eye movement measures of natural paragraph reading. Psychon. Bull. Rev..

[81] Whitford V., Titone D. (2014). The effects of reading comprehension and launch site on frequency-predictability interactions during paragraph reading. Q. J. Exp. Psychol. Hum. Exp. Psychol..

[82] Whitford V., Titone D. (2017). The effects of word frequency and word predictability during first- and second-language paragraph reading in bilingual older and younger adults. Psychol. Aging.

[83] Whitford V., Titone D. (2017). Lexical entrenchment and cross-language activation: Two sides of the same coin for bilingual reading across the adult lifespan. Biling. Lang. Cogn..

[84] SR Research Ltd (2011). EyeLink Experiment Builder.

[85] Van Patten B., Keating G.D., Leeser M.J. (2012). Missing verbal inflections as a representational problem: Evidence from self-paced reading. Linguist. Approaches Biling..

[86] SR Research Ltd (2018). EyeLink Data Viewer.

[87] SR Research Ltd (2024). EyeLink Data Viewer.

[88] Hessels R.S., Hooge I.T.C. (2019). Eye tracking in developmental cognitive neuroscience—The good, the bad and the ugly. Dev. Cogn. Neurosci..

[89] Rau A.K., Moll K., Snowling M.J., Landerl K. (2015). Effects of orthographic consistency on eye movement behavior: German and English children and adults process the same words differently. J. Exp. Child Psychol..

[90] Stevens E.A., Walker M.A., Vaughn S. (2017). The effects of reading fluency interventions on the reading fluency and reading comprehension performance of elementary students with learning disabilities: A synthesis of the research from 2001 to 2014. J. Learn. Disabil..

