# Supplementary material for: Rapid Automatized Naming (RAN) and Word Reading Fluency in Early School-Aged Children: A Pilot Eye-Tracking Study
Source: J Eye Mov Res. 2026 Feb 4;19(1):16. doi: 10.3390/jemr19010016 (PMC12921791; doi:10.3390/jemr19010016)
Supplement: Supplementary file 1 [file jemr-19-00016-s001.zip › jemr-3904554-supplementary.pdf]

Supplementary Materials

Supplementary Table S1. Data Loss by Exclusion Criteria

Reason for Data Loss	Percentage of Data Loss (of Remaining Trials)
Did not read target word	15.15%
Trials with less than 3 fixations	7.29%
Track loss	2.43%
Outliers	2.10%

Note. Percentages reflect the proportion of trials excluded at each step based on the remaining trials after the previous exclusion criterion was applied.
